# A before–after control–impact assessment to understand the potential impacts of highway construction noise and activity on an endangered songbird

**DOI:** 10.1002/ece3.2608

**Published:** 2016-12-20

**Authors:** Ashley M. Long, Melanie R. Colón, Jessica L. Bosman, Dianne H. Robinson, Hannah L. Pruett, Tiffany M. McFarland, Heather A. Mathewson, Joseph M. Szewczak, J. Cal Newnam, Michael L. Morrison

**Affiliations:** ^1^ Institute of Renewable Natural ResourcesTexas A&M UniversityCollege StationTXUSA; ^2^ Department of Wildlife and Fisheries SciencesTexas A&M UniversityCollege StationTXUSA; ^3^ Wisconsin Department of Natural ResourcesWaukeshaWIUSA; ^4^ Georgia Department of Transportation Office of Environmental ServicesAtlantaGAUSA; ^5^ Department of Wildlife, Sustainability, and Ecosystem ScienceTarleton State UniversityStephenvilleTXUSA; ^6^ Department of Biological SciencesHumboldt State UniversityArcataCAUSA; ^7^ Texas Department of TransportationAustinTXUSA

**Keywords:** before–after control–impact, construction noise, golden‐cheeked warbler, impact assessment, *Setophaga chrysoparia*, traffic noise

## Abstract

Anthropogenic noise associated with highway construction and operation can have individual‐ and population‐level consequences for wildlife (e.g., reduced densities, decreased reproductive success, behavioral changes). We used a before–after control–impact study design to examine the potential impacts of highway construction and traffic noise on endangered golden‐cheeked warblers (*Setophaga chrysoparia*; hereafter warbler) in urban Texas. We mapped and monitored warbler territories before (2009–2011), during (2012–2013), and after (2014) highway construction at three study sites: a treatment site exposed to highway construction and traffic noise, a control site exposed only to traffic noise, and a second control site exposed to neither highway construction or traffic noise. We measured noise levels at varying distances from the highway at sites exposed to construction and traffic noise. We examined how highway construction and traffic noise influenced warbler territory density, territory placement, productivity, and song characteristics. In addition, we conducted a playback experiment within study sites to evaluate acute behavioral responses to highway construction noises. Noise decreased with increasing distance from the highways. However, noise did not differ between the construction and traffic noise sites or across time. Warbler territory density increased over time at all study sites, and we found no differences in warbler territory placement, productivity, behavior, or song characteristics that we can attribute to highway construction or traffic noise. As such, we found no evidence to suggest that highway construction or traffic noise had a negative effect on warblers during our study. Because human population growth will require recurring improvements to transportation infrastructure, understanding wildlife responses to anthropogenic noise associated with the construction and operation of roads is essential for effective management and recovery of prioritized species.

## 1 Introduction

Anthropogenic noise associated with the construction and operation of highways can have individual‐ and population‐level consequences for wildlife (e.g., insects [Costello & Symes, 2014; Lampe, Reinhold, & Schmoll, 2014], amphibians [e.g., Bee & Swanson, 2007; Hoskin & Goosem, 2010], mammals [e.g., Schaub, Ostwald, & Siemers, 2008; Shier, Lea, & Owen, 2012]). Birds are the most well‐studied taxa concerning this topic, and research suggests that birds inhabiting areas near highways may be at reduced densities and have lower reproductive success (e.g., Halfwerk, Holleman, Lessells, & Slabbekoorn, 2011b; Halfwerk et al., 2011a; Reijnen & Foppen, 1991). Noise pollution along highways can also inflict hearing damage, induce physiological stress, and mask intra‐ and interspecific communications in birds (Dooling & Popper, 2007; Kaseloo, 2004; Slabbekoorn & den Boer‐Visser, 2006; Slabbekoorn & Peet, 2003; Warren, Katti, Ermann, & Brazel, 2006). In response to masking, birds may sing at higher minimum frequencies, a phenomenon termed vocal adjustment, which can require increased energy and, at the temporal extreme, could contribute to population divergence (e.g., Slabbekoorn & Smith, 2002). However, the impacts of highway construction and traffic noise on birds are species specific, and not all avifauna respond negatively (Clark & Karr, 1979; Ferris, 1979; Helldin & Seiler, 2003; Reijnen & Foppen, 2006). Therefore, it is important to quantify avian responses to noise disturbance and use the data to help guide management and regulatory actions, especially for species of conservation concern.

Determining the potential impacts of highway noise on birds can be difficult outside of laboratory conditions unless the effects are great and the potential impact is well defined. Researchers must account for (1) correlated variables (Dooling & Popper, 2007), (2) different kinds of noise occurring at the same time (e.g., vehicular traffic vs. construction activity; Burton, Armitage, Musgrove, & Rehfisch, 2002; Lackey et al., 2011; Blickley & Patricelli, 2012), and (3) variation in avian responses with increasing distance from the noise source (Halfwerk et al., 2011b; Summers, Cunnington, & Fahrig, 2011). As with other environmental impact assessments, field‐based noise studies may be limited by replication and randomization because it is rarely appropriate to replicate a potentially damaging impact, and there are few situations where assigning multiple impacts to random locations would be possible or desirable (e.g., constructing multiple roads in random locations at the same time across a variety of ecological conditions; Marshall et al., 2012). Given these limitations, researchers can employ before–after control–impact (BACI) study designs to evaluate whether a change in environmental conditions has occurred and to estimate the magnitude and duration of exposure (Morrison, Block, Strickland, Collier, & Peterson, 2008). Due to resource and time constraints, BACI designs are infrequently used to examine the potential effects of noise on birds (but see Goudie & Jones, 2004). However, the BACI framework allows researchers to address lack of replication and randomization by examining avian responses before, during, and after a noise disturbance across treatment and control sites, and to statistically evaluate potential impacts by testing for interactions between time and site variables (Green, 1979; Underwood & Chapman, 2003).

We used a BACI design to examine the potential impacts of highway construction noise on golden‐cheeked warbler (*Setophaga chrysoparia*; hereafter warbler) habitat selection, reproduction, and behavior in an urban setting. The warbler is an endangered, migratory songbird that breeds exclusively in central Texas, USA (Komar et al., 2013; Ladd & Gass, 1999; Pulich, 1976). The U.S. Fish and Wildlife Service listed the species under the U.S. Endangered Species Act in 1990, citing habitat loss and fragmentation as the primary threats to warbler persistence (USFWS 1990). These factors will likely remain a concern for warblers as human populations grow and their associated infrastructure expands to meet demand (Groce, Mathewson, Morrison, & Wilkins, 2010). Previous research suggests that highway noise does not affect the presence, productivity, density, or behavior of warblers in rural locations (Benson, 1995; Lackey et al., 2011; Mathewson et al., 2013), but warbler responses to anthropogenic noise in louder, urban settings are unknown.

We mapped and monitored warbler territories before (2009–2011), during (2012–2013), and after (2014) highway construction at (1) a treatment site exposed to highway construction and traffic noise, (2) a control site exposed only to traffic noise, and (3) a control site exposed to neither highway construction or traffic noise. We recorded noise at varying distances from the highways at our construction and traffic noise sites. We expected that noise would be greatest at the construction site during the years of construction and that noise would decrease with increasing distance from the highway at both sites. If warblers responded negatively to highway construction noise, we predicted they would exhibit one or more of the following responses at the construction site during‐ or postconstruction relative to the preconstruction period: reduced densities, displacement from the highway, decreased pairing or fledging success, or higher minimum song frequencies. In addition, we predicted that warblers at the control site and those farther from highways would exhibit acute responses to experimentally introduced highway construction noise as individual birds located further from the sound source would be experiencing a novel disturbance. The results of our comprehensive assessment may help inform conservation efforts for warblers. In addition, our study demonstrates the use of a robust BACI study design to quantify wildlife responses to anthropogenic noise.

## 2 Materials and Methods

### 2.1 Study area and design

**Figure 1:**
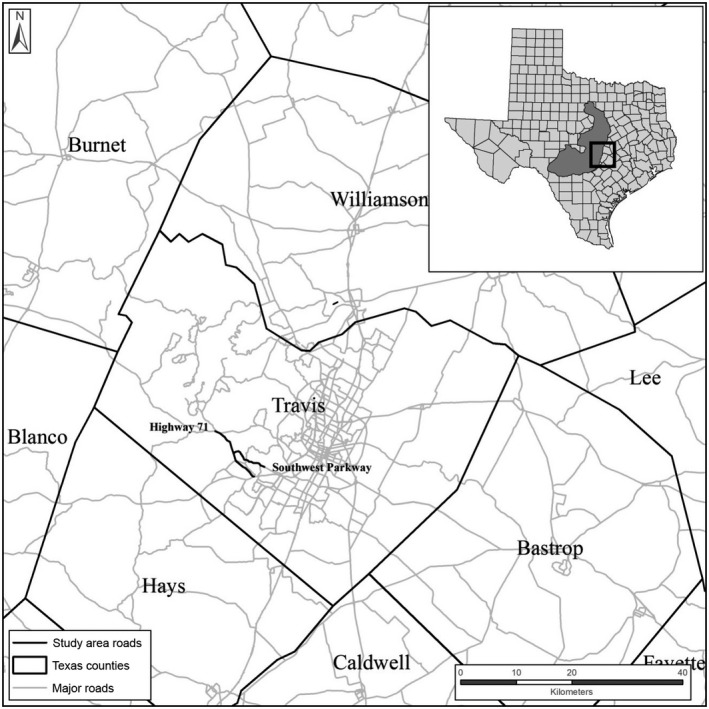
Study area along Highway 71 and Southwest Parkway in Austin, Texas, USA, for research examining the impacts of road noise on golden‐cheeked warblers (*Setophaga chrysoparia*) from 2009 to 2014. Inset shows the warbler's breeding range

We conducted our research from March to July 2009–2014 in Austin, Texas (~30°N, 98°W), located on the eastern edge of the warbler's breeding range (Figure 1). Warbler habitat in this region is typically composed of Ashe juniper (*Juniperus ashei*), Texas oak (*Quercus buckleyi*), live oak (*Q. virginiana*), and various other hardwoods (Diamond, 1997). Existing habitat in the city of Austin is fragmented by urban and residential development (Collier et al., 2012; Mathewson et al., 2012). Local mean minimum and mean maximum temperatures in July are 27°C and 35°C (NOAA 2015). Mean annual precipitation is 87 cm (NOAA 2015).

We delineated one treatment site and two types of control sites at the Barton Creek Habitat Preserve, a 1,600‐ha property managed by The Nature Conservancy. We selected study sites with similar ecological characteristics that were exposed to similar environmental conditions throughout the year (e.g., temperature, precipitation). The treatment site (hereafter construction site) was a 301‐ha area of potential warbler habitat located ≤800 m from a 5.2 km segment of Highway 71 that was exposed to construction noise associated with highway expansion in 2012 and 2013 and traffic noise throughout the study. Annual average daily traffic (AADT) rates for this portion of the highway were 30,000–39,000 vehicles/day (TXDOT 2012). As such, AADT on Highway 71 was comparable to traffic loads on highways located in other large U.S. cities (e.g., Houston, Dallas; TXDOT 2016). During the Highway 71 expansion project, the Texas Department of Transportation did not remove warbler habitat along the highway, but warblers were exposed to noises associated with construction activities, such as backup warning beepers, diesel engine noise, and loading dump trucks. The first of our control sites (hereafter traffic noise site) was a 416‐ha area of potential warbler habitat located ≤800 m from a 2.8‐km segment of Southwest Parkway adjacent to Highway 71 (Figure 1) that did not undergo construction during our study and, therefore, allowed us to separate the effects of traffic noise from construction noise on warbler responses. AADT rates for this portion of Southwest Parkway were 16,200 vehicles/day (C. Newnam, *pers. comm*.). Our second control site (hereafter control site) included 682‐ha of potential warbler habitat located >800 m from Highway 71 or other major roads. We collected data at all sites during three treatment phases: preconstruction (2009–2011), construction (2012–2013), and postconstruction (2014).

### 2.2 Noise

We established paired transects perpendicular to Highway 71 in the construction site and perpendicular to Southwest Parkway in the traffic noise site. We spaced transect pairs across the construction and traffic noise sites to account for potential within‐site variability in warbler density. As such, the number of transect pairs (two or three) depended on the total area of each study site. We placed Extech 407764 Datalogging Sound Meters (Extech Instruments, Nashua, New Hampshire, USA) programmed to record noise from 06:00 to 12:00 on each transect at stations 16, 32, 64, 128, 256, and 512 m from the highways. We randomly sampled one transect pair every 2 days from late March to late May, rotating between the construction and traffic noise sites. This protocol allowed us to sample 2–3 transect pairs per week throughout much of the warbler breeding season during the hours when songbirds are most active. We calculated the mean and maximum noise for each sound meter for use in analyses. We did not place sound meters at the control site because pilot data collected in 2008 indicated that highway noise could not be detected at distances >800 m from highways in the study area. During the pilot year, mean noise levels were 36 dB(A) at the control site, which is consistent with noise levels recorded in libraries and quiet rural areas.

### 2.3 Territory density, placement, and size

We established transects ≥300 m apart covering the spatial extent of each study site. Transects in the construction and traffic noise sites extended ≤1 km from the highways. Transects in the control site were ≤1.5 km and located ≥800 m from the nearest highway. Each day from late February to late March, we slowly walked (~1 km/h) along transects from 07:00 to 13:00 and recorded the locations of singing male warblers using handheld Global Positioning System (GPS) units (≤10 m accuracy). If we heard another warbler in close proximity to our initial detection, we walked to the newly identified bird and recorded a GPS point at its location. After we obtained GPS locations on all singing males in the area, we returned to the transect to search for additional warblers. We used this methodology to ensure full survey coverage of the study sites, but we did not use detections recorded during transect surveys for analyses purposes. Rather, we used the locations to aid relocation of male warblers during subsequent territory mapping and monitoring activities.

We mapped and monitored each territory for up to 1 hr at least once per week for the duration of the warbler breeding season (March–June). If we detected previously unidentified individuals during our territory mapping and monitoring surveys, we recorded GPS locations for the new individuals and added the territories to our weekly survey rotation. Our intent with this methodology was to identify all territorial males within each study site.

During territory mapping in 2009–2010, we recorded GPS locations of males each time they moved ≥20 m until we recorded 3–6 locations per sampling occasion. In 2011, we modified our territory mapping protocols and recorded GPS locations of males every 2 min for 1 hr or until we could no longer detect focal individuals (Barg, Jones, & Robertson, 2005). We used data collected by both methods to create minimum convex polygons (MCPs; the outermost points in a location dataset) for all territories with ≥15 point locations and identified the associated centroids (i.e., the central point in each MCP) for each territorial male (i.e., male relocated for ≥4 weeks). We stopped mapping territories once we detected fledglings or once we were unable to detect the focal male during three consecutive visits. Because we repeatedly and thoroughly covered each study site during our surveys, we defined territory density as the number of MCPs per hectare of potential warbler habitat within each study site. We defined territory placement as the distance of the territory from the highway, which we measured as the shortest distance of each territory centroid from the highway in the construction and traffic noise sites. We only included warbler territories that were located ≤400 m from highways in our distance analyses to ensure that territories were plausibly exposed to construction or traffic noise (see Noise section above; Reijnen & Foppen, 2006; Summers et al., 2011; McClure, Ware, Carlisle, Kaltenecker, & Barber, 2013).

### 2.4 Pairing and fledging success

We used a modified version of the Vickery Index (Vickery, Hunter, & Wells, 1992) to examine the reproductive status of each territory, specifically the male's pairing and fledging success (as in Klassen, Morrison, Mathewson, Rosenthal, & Wilkins, 2012; Marshall, Morrison, & Wilkins, 2013; Stewart, Morrison, Hutchinson, Appel, & Wilkins, 2014). We observed warbler behavior and activity during each territory mapping visit and ranked the status of the territory as follows: (1) male present ≥4 weeks, (2) pair present ≥4 weeks, (3) material carried to the presumed nest, (4) food carried to the presumed nestlings, and (5) fledglings sighted by the observer. When the behavioral rank recorded in a territory was ≥2, we considered the male successfully paired. When we recorded a behavioral rank of five, we considered the pair reproductively successful. We calculated pairing success as the number of successfully paired territorial males relative to the total number of territorial males. We calculated fledging success as the number of paired territorial males that successfully fledged one or more host young relative to the total number of paired territorial males.

Direct measures of nest success would provide more detailed analyses of reproductive output (Reidy, O'Donnell, & Thompson, 2015). However, reproductive indices, such as the Vickery method, allow observers to avoid potential biases associated with nonrandomly collected nest data (Martin & Geupel, 1993), sample a larger spatial extent (Villard & Pärt, 2004), and predict territory outcomes when females or nests are difficult to locate and monitor (Craft, 1998). Additionally, the Vickery method limits disruption of nesting pairs (Götmark, 1992; Maas, 1998), which is important for studies that involve rare or endangered species. Warblers maintain territorial boundaries during the breeding season (Ladd & Gass, 1999), but there can be overlap around territory edges. We designed our methods such that incursions of neighboring warblers into adjacent territories did not negatively affect our ability to assign productivity outcomes to territories. We took extreme care to properly link breeding outcomes with specific territories by conducting repeated visits to each territory over the course of the breeding season and only assigning fledglings ≤2 weeks of age to territories. Because we used the same methodology across sites, we are confident in assuming that any error in assigning reproductive outcomes to territories was similar across sites.

### 2.5 Song characteristics

Each year of the study, we placed Song Meter SM2 digital field automatic recording units (ARUs; Wildlife Acoustics, Maynard, Massachusetts, USA) across all study sites to examine how highway construction and traffic noise influenced warbler song characteristics. We placed ARUs in randomly selected territories at various distances from the highways (0–300, 300–600, 600–900 m) at the construction and traffic noise sites. We placed ARUs randomly within the control site given a lack of linear features comparable to the highways at other sites. We programmed ARUs to record from ~06:45 to 11:00 daily. We allowed each ARU to record warbler songs for 3–4 weeks before moving it to another randomly selected territory.

We used SonoBird^™^ v1.5.8 (DNDesign, Arcata, CA, USA) to extract warbler songs from our recordings. The extraction process identified warbler songs using time–frequency patterns within the waveforms of the recordings but also identified songs of other species that fell within the same frequency range (e.g., black‐throated green warblers [*S. virens*] and northern cardinals [*Cardinalis cardinalis*]). Therefore, we visually inspected the sonograms of extracted songs to ensure proper species identification. We also identified and excluded any sonograms with extensive background noise that could interfere with subsequent calculations of warbler song metrics (e.g., warbler song with northern cardinal singing in the background).

We used SonoBird^™^ v1.6.5 (DNDesign, Arcata, CA, USA) to manually analyze warbler songs. Warblers have two primary song types, the A‐song and the B‐song (Bolsinger, 2000), and each type is divided into three phrases. We identified each individual song by type and phrase. Within each phrase, we obtained lower and upper bandwidth cutoffs across phrase‐specific time steps to represent the mean lower frequency and mean upper frequency. The difference between the two bounds represented the bandwidth of the phrase.

### 2.6 Playback experiment

We conducted a playback experiment within a subset of territories to examine warbler responses to played recordings of construction noise at ~80 dB(A) (measured at ~5 m)—a level known to be annoying to humans but that does not cause hearing damage (Ristovska, Gjorgjev, Polozhani, Kočubovski, & Kendrovski, 2009). The primary noises of the treatment broadcast included backup warning beeps, diesel engine noise, and loading dump trucks, and we used the same noise clip in all surveys. To control for the potential effects of observer presence, we also replicated the methodology of the playback surveys without playing the construction noise recordings. We conducted our playback surveys on days with and without construction activity, but we did not broadcast construction noise more than once every 10 days to avoid habituating individuals to the recordings.

We randomly selected warbler territories in all sites at varying distances from the highways to receive a construction or control playback. Once we located a male warbler in the selected territory, we recorded the individual's behavior for 2 min. We then broadcast construction noise with a handheld speaker or displayed a silent handheld speaker to the warbler for a maximum of 5 s. During playback surveys, we maintained a distance of ~20 m from the focal bird to limit observer influence on the warbler's response. Each construction or control playback ceased after 5 s or as soon as the subject's behavior changed. We considered a playback experiment to have elicited a behavioral response if the warbler stopped singing or flew from its previous perch and out of the surveyor's view (≥10 m). We recorded the presence or absence of a behavioral response to the construction or control playback.

### 2.7 Analyses

For an impact assessment using the BACI framework, data analyses should include an interaction term between treatment site and phase to determine whether there is a statistically significant difference at the treatment site following the disturbance (Morrison et al., 2008:229–264). For this study, a change in any of the metrics of interest at the construction site during or after the construction phase relative to other phases and sites—as represented by a statistically significant interaction—would suggest that construction activities had affected warblers. For example, to find evidence in support of the vocal adjustment hypothesis under the BACI framework, higher minimum frequency songs recorded at the construction site during‐ or postconstruction relative to the preconstruction phase could indicate that warblers adjusted their communication signals to avoid masking (reviewed in Patricelli & Blickley, 2006) and, therefore, could suggest that a construction noise‐related impact had occurred.

We used two‐way factorial analyses of variance (ANOVA) to test the interactive effects of site, treatment phase, and distance from the highway on our continuous response variables (i.e., noise, song metrics). We used logistic regression to examine the interactive effects of site and phase on our binary response variables (i.e., pairing and fledging success). If there was a significant interaction (α = 0.05), we visually examined plots with calculated means and 95% confidence intervals (hereafter 95% CI) to determine the direction, magnitude, and biological significance of the patterns. Sample sizes or other factors sometimes required that we deviate from the general approach described above. We used a nonparametric Friedman's rank‐sum test to examine territory density in relation to site and phase (Zar, 1999). Friedman's test is preferred over ANOVA with repeated measures when there is only one observation for the response variable in each combination of levels of groups and blocks and where the normality assumption may be violated (Zar, 1999). In our case, we had one measure of territory density for each site and phase combination. Additionally, low sample sizes prohibited us from examining the interactive effects of site, phase, and distance from the roadway on territory placement within 400 m of highways. Instead, we used linear regression to examine how distance from the highway affected territory placement separately by site and phase. We did not examine warbler responses as a function of distance at our control site because the minor roads and trails within the site were seldom used and did not resemble linear edges comparable to Highway 71 or Southwest Parkway. We used logistic regression to determine whether survey type or site independently influenced warbler responses during our playback experiments. We then used logistic regression to determine whether the probability of a warbler response to experimental playback recordings increased with increasing distance from highways. We performed all statistical analyses using the open‐source program R v. 3.2.2 (R Core Development Team, Vienna, Austria).

## 3 Results

### 3.1 Noise

**Figure 2:**
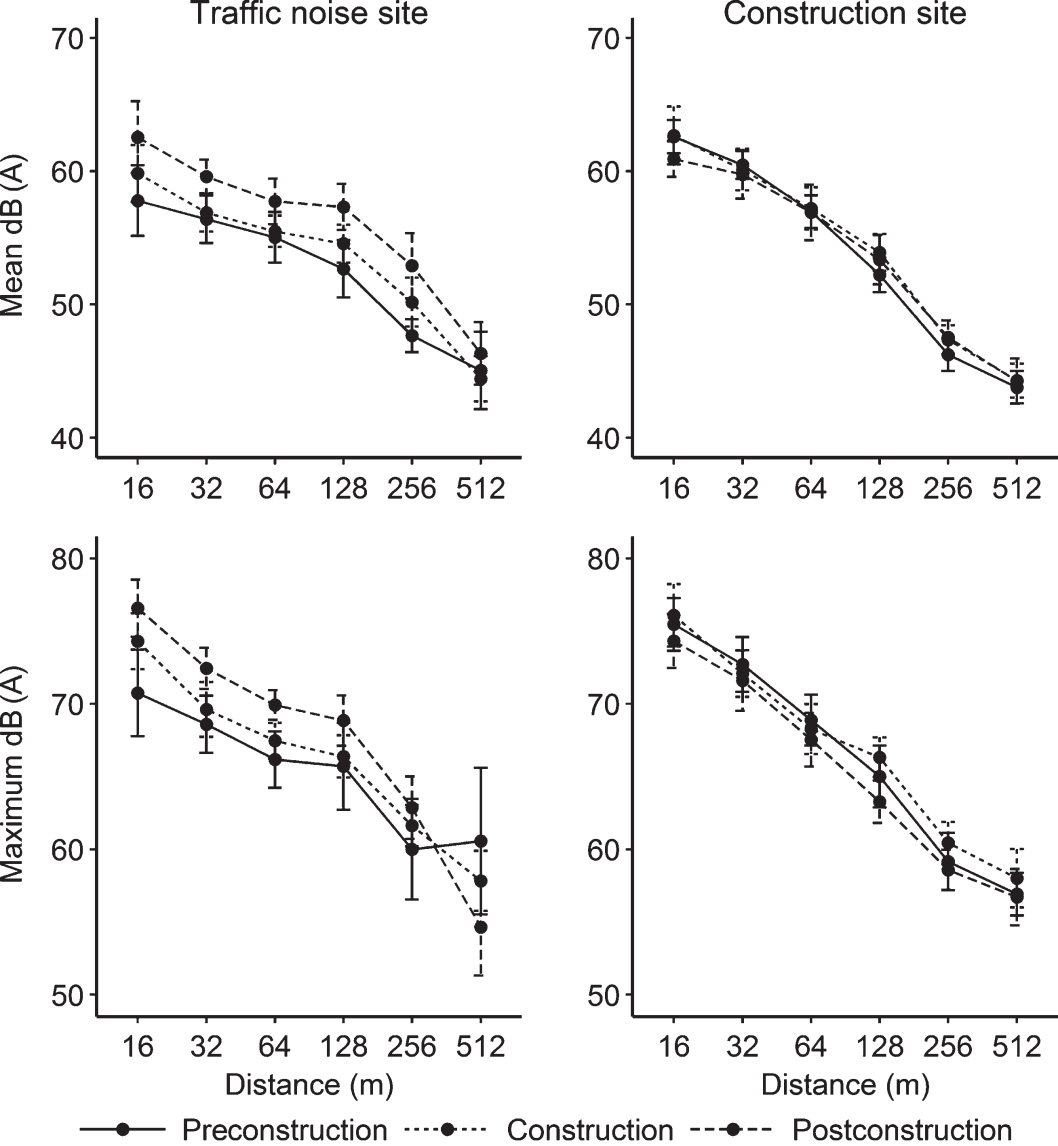
Mean and maximum noise and associated 95% confidence intervals recorded in golden‐cheeked warbler (*Setophaga chrysoparia*) habitat at varying distances from the road before (2009‐2011), during (2012‐2013), and after (2014) construction activity in Austin, Texas, USA

We found a statistically significant interaction between distance and site (*F*_5,883_ = 9.81, *p < *.01), indicating that differences in noise levels across the distance from highway categories depended on whether the sound meters were located in the construction site or traffic noise site. As such, we examined noise across treatment phases separately by site. Mean noise levels were 54 dB(A) ± 8 *SD* in the construction site and 53 dB(A) ± 6 *SD* in the traffic noise site. Mean maximum noise levels were 67 dB(A) ± 8 *SD* in the construction site and 66 dB(A) ± 7 *SD* in the traffic noise site. Mean and maximum noise decreased by ≤8 dB(A) with each increasing distance category from the highway across all three treatment phases at both the construction and traffic noise sites (Figure 2). We did not find a statistically significant interaction between treatment phase and distance from the highway on mean noise in the construction site (*F*_10,561_ = 0.57, *p = *.84) or in the traffic noise site (*F*_10,298_ = 0.65, *p = *.77). Similarly, we did not find a statistically significant interaction between treatment phase and distance from the highway on maximum noise in the construction site (*F*_10,561_ = 0.41, *p = *.94). However, we did find a statistically significant interaction at the traffic noise site (*F*_10,298_ = 2.36, *p< *.01). Mean and maximum noise at distances closest to the highway in the traffic noise site were ≤10 dB(A) higher during the construction and postconstruction phases when compared to the preconstruction phase. However, the substantially overlapping 95% CIs indicated that the differences in noise were not statistically different across the treatment phases for most of the distance categories at either site (Figure 2).

### 3.2 Territory density and placement

We mapped and monitored 450 warbler territories across the years of this study (Table 1). We found a significant difference in territory density across treatment sites and phases (Friedman χ_2_^2^ = 6.00, *p *=* *.05). Mean territory density was 1.3 to 1.8 times greater in the control site than the construction and traffic noise sites (Table 2). Mean territory density was between 1.5 and 1.7 times greater during the postconstruction phase than during the preconstruction phase (Table 2). We found no difference in mean territory distance from the highways (Table 2) across sites (*F*_1,67_ = 0.36, *p *=* *.55) or phases (*F*_2,66_ = 1.29, *p *=* *.28).

**Table 1:** Summary of cumulative pairing and fledging success in monitored territories by treatment site and phase used to examine the potential impacts of highway construction noise on golden‐cheeked warblers (*Setophaga chrysoparia*) in Austin, Texas, USA (2009–2014). Construction occurred from 2012 to 2013

Treatment phase^a^	Site	Monitored territories	Pairing success^b^		Fledging success^c^	
		#	#	%	#	%
Preconstruction	Construction	39	33	85	26	79
	Traffic noise	42	33	79	22	67
	Control	105	75	71	54	72
Construction	Construction	29	21	72	13	62
	Traffic noise	40	27	68	18	67
	Control	101	75	74	57	76
Postconstruction	Construction	19	13	68	10	77
	Traffic noise	23	21	91	13	62
	Control	52	38	73	29	76

a Preconstruction = 2009‐2011, construction = 2012‐2013, and postconstruction = 2014.

b Territories defined as paired if a female was present for ≥4 weeks.

c Territories defined as fledged if ≥1 host young fledged from any nest attempt within a territory.

**Table 2:** Mean golden‐cheeked warbler (*Setophaga chrysoparia*) territory densities with standard deviations in parentheses and mean distance with 95% confidence intervals in parentheses for territories ≤400 m from roads by treatment site and phase in Austin, Texas, USA

Treatment phase^a^	Site	Density^b^^,^ c	Distance (m)^d^
Preconstruction	Construction	0.04 (0.00)	291.64 (63.58)
	Traffic noise	0.03 (0.01)	238.84 (43.57)
	Control	0.05 (0.01)	NA
Construction	Construction	0.05 (0.00)	243.14 (97.70)
	Traffic noise	0.04 (0.01)	229.92 (67.40)
	Control	0.07 (0.01)	NA
Postconstruction	Construction	0.06	204.82 (69.63)
	Traffic noise	0.06	218.13 (104.77)
	Control	0.08	NA

a Preconstruction = 2009‐2011, construction = 2012‐2013, and postconstruction = 2014.

b Territories/ha.

c Standard deviation not applicable for postconstruction phase with only 1 year of data.

d Distance to the roadway not applicable at the control site.

### 3.3 Pairing and fledging success

Pairing success and fledging success ranged from 68% to 91% and 62% to 79%, respectively, during the three phases of our study (Table 1). We found no statistically significant interaction between site and phase on warbler pairing success (χ_4_^2^ = 7.14, *p *=* *.13). We also found no statistically significant interaction between site and phase on warbler fledging success (χ_4_^2^ = 2.42, *p *=* *.66).

### 3.4 Song characteristics

We analyzed >19,000 A‐songs (*n *=* *72 song meters) and >3,500 B‐songs (*n *=* *48 song meters). Sample sizes were low for B‐songs recorded during the postconstruction phase, so we were unable to examine statistically significant interactions for any of the B‐song metrics. As such, we excluded these data from this study. We found no statistically significant interactions between treatment site and phase for most A‐song metrics (Tables 3 and 4), and differences that were statistically significant were unrelated to construction noise. Similarly, we found no statistically significant interactions between treatment phase and distance from the highway (0–300, 300–600, 600–900, and >900 m) for most A‐song metrics (Tables 3 and 5). Again, statistically significant differences were unrelated to construction noise.

**Table 3:** Results for two‐way factorial analyses of variance (ANOVA) of A‐song metrics (kHz) for a study examining the potential impacts of highway construction noise on golden‐cheeked warblers (*Setophaga chrysoparia*) in Austin, Texas, USA (2009–2014)

Analysis^a^	Phrase	Lower frequency	Upper frequency	Bandwidth
Site*phase	1	*F*_4,106_ = 0.66, *p* = .62	*F*_4,106_ = 1.06, *p* = .38	*F*_4,106_ = 0.78, *p* = .54
	2	*F*_4,110_ = 0.17, *p* = .95	*F*_4,110_ = 3.35, *p* = .01	*F*_4,110_ = 2.67, *p* = .04
	3	*F*_4,109_ = 1.81, *p* = .13	*F*_4,109_ = 0.58, *p* = .68	*F*_4,109_ = 1.11, *p* = .36
Dist*phase	1	*F*_6,103_ = 2.75, *p* = .02	*F*_6,103_ = 0.43, *p* = .86	*F*_6,103_ = 1.77, *p* = .11
	2	*F*_6,107_ = 0.92, *p* = .48	*F*_6,107_ = 1.62, *p* = .15	*F*_6,107_ = 2.17, *p* = .05
	3	*F*_6,106_ = 1.55, *p* = .17	*F*_6,106_ = 0.78, *p* = .59	*F*_6,106_ = 0.15, *p* = .99

a Interaction between site (construction, traffic noise, and control) and treatment phase (preconstruction [2009‐2011], construction [2012‐2013], and postconstruction [2014]) and interaction between distance from road (0–300, 300–600, 600–900, and >900 m) and treatment phase.

**Table 4:** Means and associated 95% confidence intervals in parentheses for lower frequency, upper frequency, and bandwidth of A‐songs per treatment site and phase used to examine the potential impacts of highway construction on golden‐cheeked warblers (*Setophaga chrysoparia*) in Austin, Texas, USA (2009–2014)

Treatment phase^a^	Phrase	Metric (kHz)	Construction	Traffic noise	Control
Preconstruction	1	Lower frequency	3.87 (0.13)	3.75 (0.14)	3.76 (0.17)
		Upper frequency	6.13 (0.13)	6.13 (0.09)	5.98 (0.19)
		Bandwidth	2.25 (0.19)	2.38 (0.19)	2.22 (0.12)
	2	Lower frequency	4.30 (0.09)	4.28 (0.10)	4.22 (0.11)
		Upper frequency	6.56 (0.09)	6.58 (0.07)	6.33 (0.16)
		Bandwidth	2.27 (0.10)	2.30 (0.11)	2.10 (0.10)
	3	Lower frequency	6.23 (0.10)	6.15 (0.06)	6.18 (0.08)
		Upper frequency	7.64 (0.11)	7.67 (0.16)	7.61 (0.11)
		Bandwidth	1.41 (0.08)	1.52 (0.17)	1.44 (0.15)
Construction	1	Lower frequency	3.94 (0.18)	4.02 (0.15)	3.87 (0.14)
		Upper frequency	6.16 (0.09)	6.20 (0.13)	6.19 (0.11)
		Bandwidth	2.22 (0.19)	2.18 (0.21)	2.32 (0.15)
	2	Lower frequency	4.35 (0.06)	4.33 (0.10)	4.31 (0.09)
		Upper frequency	6.51 (0.07)	6.61 (0.12)	6.53 (0.11)
		Bandwidth	2.15 (0.08)	2.28 (0.14)	2.22 (0.10)
	3	Lower frequency	6.18 (0.09)	6.22 (0.09)	6.17 (0.10)
		Upper frequency	7.65 (0.15)	7.55 (0.15)	7.62 (0.15)
		Bandwidth	1.47 (0.13)	1.33 (0.14)	1.44 (0.16)
Postconstruction	1	Lower frequency	4.00 (0.16)	4.06 (0.20)	3.99 (0.13)
		Upper frequency	6.11 (0.15)	6.31 (0.27)	6.15 (0.18)
		Bandwidth	2.11 (0.16)	2.25 (0.38)	2.16 (0.17)
	2	Lower frequency	4.32 (0.14)	4.36 (0.11)	4.30 (0.09)
		Upper frequency	6.35 (0.08)	6.57 (0.19)	6.50 (0.14)
		Bandwidth	2.03 (0.13)	2.21 (0.11)	2.20 (0.15)
	3	Lower frequency	6.06 (0.09)	6.13 (0.13)	6.21 (0.11)
		Upper frequency	7.56 (0.13)	7.57 (0.23)	7.67 (0.29)
		Bandwidth	1.50 (0.12)	1.44 (0.12)	1.46 (0.22)

a Preconstruction = 2009‐2011, construction = 2012‐2013, and postconstruction = 2014.

**Table 5:** Means and associated 95% confidence intervals for selected A‐song metrics per treatment phase and distance from the road used to examine the potential impacts of highway construction on golden‐cheeked warblers (*Setophaga chrysoparia*) in Austin, Texas, USA (2009–2014)

Treatment phase^a^	Phrase	Metric (kHz)	Distance (m)	Mean (95% CI)
Preconstruction	1	Lower frequency	0–300	3.74 (0.20)
			600–900	3.88 (0.16)
	2	Bandwidth	0–300	2.35 (0.11)
			600–900	2.29 (0.14)
Construction	1	Lower frequency	0–300	4.19 (0.14)
			600–900	3.79 (0.24)
Postconstruction	1	Lower frequency	300–600	4.19 (0.31)

a Preconstruction = 2009‐2011, construction = 2012‐2013, and postconstruction = 2014.

### 3.5 Playback experiment

We conducted 321 experimental playback surveys and 96 control surveys within 172 warbler territories. Playback surveys ranged from 48 to 1,900 m from the highway. In the construction site, six warblers (10%) responded to experimental playback and one warbler (~6%) responded to control surveys. In the traffic noise site, five warblers (~6%) responded to experimental playback and one warbler (~5%) responded to control surveys. In the control site, 20 warblers (~11%) responded to experimental playback and two warblers (~3%) responded to control surveys. We found no significant main effect of playback survey type (χ_1_^2^ = 3.30, *p *=* *.07) or site (χ_2_^2^ = 0.90, *p *=* *.64) on warbler responses, and the predicted probability of a warbler response to experimental playback recordings of construction noise did not increase with increasing distance from Highway 71 or Southwest Parkway (χ_1_^2^ = 0.03, *p *=* *.86).

## 4 Discussion

Warbler territory density increased over time at all study sites, and we found no differences in warbler territory placement, productivity, song characteristics, or behavior that we could attribute to the highway construction or traffic noise that occurred during our study. Noise levels recorded at our study sites were similar or higher when compared to those recorded within warbler habitat at rural locations (Benson, 1995; Mathewson et al., 2013), and previous research suggests that some bird species are sensitive to noise levels we recorded at our study sites (e.g., Forman, Reineking, & Hersperger, 2002; Kaseloo, 2004; Reijnen, Foppen, & Veenbaas, 1997). However, the noise levels we recorded in Austin, Texas, were similar before, during, and after construction at the construction site, and noise levels were similar at the construction and traffic noise sites (Figure 2). As such, we would only expect to find differences in our warbler responses between these sites if there was an unmeasured source of anthropogenic disturbance associated with construction or traffic (e.g., visual disturbance, vibrations, dust) that negatively impacted the birds or if there were ecological differences across study sites that we did not account for in our study design. Given similarities in warbler responses across all treatment phases and sites and with increasing distance from the highways, there is no evidence to support these alternative hypotheses.

It is possible that greater levels of highway construction or traffic noise could have a negative impact on warblers. However, the results of our playback study where we exposed birds to recordings of construction noise at ~80 dB(A) suggest that the noise would have to be close range and much higher than levels typically recorded at construction sites (e.g., 90 dB(A); Kerr, Brousseau, & Johnson, 2002). Alternatively, warblers may be more responsive to construction noise that is chronic. Previous research demonstrated that yellow‐rumped warblers (*S. coronata*) are less likely to place their territories in close proximity to compressors used in oil and gas production, which typically operate at 75–90 dB(A) (maximum 105 dB(A)), 24 hr a day, 365 days a year (Bayne, Habib, & Boutin, 2008). Similarly, ovenbirds (*Seiurus aurocapilla*) exhibit reduced pairing success when exposed to sustained oil and gas compressor noise (Habib, Bayne, & Boutin, 2006). Golden‐cheeked warblers at our construction and traffic noise sites experienced noise levels similar to those produced by oil and gas compressors, but only periodically, so we cannot address this topic without experimentally introducing warblers to louder, more sustained construction noise, an exercise unlikely to mimic a plausible disturbance in warbler habitat.

The frequency of anthropogenic noise that birds are exposed to may be more influential than amplitude (Dooling & Popper, 2007). Typical traffic noise ranges from 1 to 2 kHz (Blickley & Patricelli, 2012; Dooling & Popper, 2007; Warren et al., 2006), which overlaps with the song frequencies of many, but not all, songbirds (Morton, 1975; Rheindt, 2003). Species, like the golden‐cheeked warbler, that forage in the upper portions of the canopy often sing at higher frequencies (Ficken & Ficken, 1962; Lemon, Struger, Lechowicz, & Norman, 1981) and may be less susceptible to vocal masking. For example, the yellow‐rumped warbler, a congeneric species that inhabits similar ecological conditions, has life‐history characteristics comparable to the golden‐cheeked warbler, and sings from ~3 to 7 kHz, avoids chronic industrial noise but not roads (Ware, McClure, Carlisle, & Barber, 2015). These species can likely hear low‐frequency traffic noise, but because they communicate at higher frequencies, individuals may be unaffected by the sound. The golden‐cheeked warbler's song ranges from 3 to 9 kHz, but is typically between 4 and 8 kHz (Bolsinger, 2000), which may explain why we did not observe negative responses to noise associated with highway construction or traffic. We did find a small number of statistically significant differences in certain song characteristics, but these were not related to construction or traffic noise and are likely attributable to individual variation.

The USFWS previously indicated that noise levels occurring at ≥60 dB(A) during the loudest hour of the day may negatively influence songbirds (Barrett, 1995; Dooling & Popper, 2007). However, the USFWS also recognizes that species respond differently to noise depending on their life‐history traits and the level and persistence of noise exposure. As such, noise levels are considered high when they exceed 10 dB(A) over background noise (Patricelli, Blickley, & Hooper, 2012). Although maximum noise occasionally exceeded 60 dB(A) nearest to the highways at our construction and traffic noise sites, mean noise levels were ~60 dB(A) closest to the roads and decreased with increasing distance from Highway 71 and Southwest Parkway (Figure 2). Moreover, the average maximum noise at our construction and traffic noise sites was only ~5 dB(A) greater than the average maximum noise recorded at a rural control site during a similar study where we also found no evidence to suggest that construction noise and activity had a negative effect on warblers (M. L. Morrison, *unpublished data*).

Anthropogenic noise associated with highway construction and operation can have individual‐ and population‐level consequences for wildlife. However, the impacts of highway noise on birds are species specific (reviewed in Reijnen & Foppen, 2006). As such, the potential impacts to individual species should be experimentally tested rather than assumed. We found no quantitative evidence that highway construction and traffic noise at our sites had a negative effect on warblers, but many other factors can influence warbler abundance and productivity (e.g., patch size: Arnold, Coldren, & Fink, 1996; Baccus, Tolle, & Cornelius, 2007; Butcher, Morrison, Ransom, Slack, & Wilkins, 2010; Robinson, 2013; landscape composition: Collier et al., 2012; Mathewson et al., 2012; edge‐to‐area ratio: Peak, 2007; tree species composition: Marshall et al., 2013; Long, 2014; canopy cover: Dearborn & Sanchez, 2001; Magness, Wilkins, & Hejl, 2006; age: Jette, Hayden, & Cornelius, 1998; Pruett, 2014; presence of conspecifics: Farrell, Morrison, Campomizzi, & Wilkins, 2012), and there is evidence to suggest that warblers are more sensitive to these explanatory variables in urban settings (Robinson, 2013). Research that examines these potential constraints, especially in light of habitat loss and conversion, may promote more effective management and recovery of the species. Human population growth will require recurring improvements to transportation infrastructure. We encourage the use of a robust BACI design, or similar experimental field‐based approaches, to quantitatively assess the potential impacts of highway construction on wildlife, the results of which can be used to support conservation of prioritized species.

## Conflict of Interest

None declared.
